# Supplementary material for: Imprinted SARS-CoV-2 humoral immunity induces convergent Omicron RBD evolution
Source: Nature. 2022 Dec 19;614(7948):521–9. doi: 10.1038/s41586-022-05644-7 (PMC9931576; doi:10.1038/s41586-022-05644-7)
Supplement: Supplementary file 1 — Reporting Summary [file 41586_2022_5644_MOESM1_ESM.pdf]

nature portfolio

Corresponding author(s): Yunlong Cao, Youchun Wang, Xiaoliang Sunney Xie

Last updated by author(s): Dec 6, 2022

Reporting Summary

Nature Portfolio wishes to improve the reproducibility of the work that we publish. This form provides structure for consistency and transparency in reporting. For further information on Nature Portfolio policies, see our Editorial Policies and the Editorial Policy Checklist.

Statistics

For all statistical analyses, confirm that the following items are present in the figure legend, table legend, main text, or Methods section.

n/a	Confirmed	
☐	☒	The exact sample size (*n*) for each experimental group/condition, given as a discrete number and unit of measurement
☐	☒	A statement on whether measurements were taken from distinct samples or whether the same sample was measured repeatedly
☐	☒	The statistical test(s) used AND whether they are one- or two-sided<br>*Only common tests should be described solely by name; describe more complex techniques in the Methods section.*
☒	☐	A description of all covariates tested
☒	☐	A description of any assumptions or corrections, such as tests of normality and adjustment for multiple comparisons
☐	☒	A full description of the statistical parameters including central tendency (e.g. means) or other basic estimates (e.g. regression coefficient) AND variation (e.g. standard deviation) or associated estimates of uncertainty (e.g. confidence intervals)
☐	☒	For null hypothesis testing, the test statistic (e.g. *F*, *t*, *r*) with confidence intervals, effect sizes, degrees of freedom and *P* value noted<br>*Give P values as exact values whenever suitable.*
☒	☐	For Bayesian analysis, information on the choice of priors and Markov chain Monte Carlo settings
☒	☐	For hierarchical and complex designs, identification of the appropriate level for tests and full reporting of outcomes
☒	☐	Estimates of effect sizes (e.g. Cohen's *d*, Pearson's *r*), indicating how they were calculated

Our web collection on statistics for biologists contains articles on many of the points above.

Software and code

Policy information about availability of computer code

Data collection

Pseudovirus neutralization and ELISA data were collected by microplate spectrophotometer (PerkinElmer, HH3400).
FACS data was collected by Summit 6.0 (Beckman Coulter).

Data analysis

Neutralization assays data were analyzed using PRISM (v9.0.1) .
FACS data were analyzed by FlowJo 10.8.
Sequence alignment of Omicron sublineages was performed by biopython (v1.78); V(D)J sequence data were analyzed using Cell Ranger (v6.1.1), IgBlast (v1.17.1). Somatic hypermutation sites in the antibody variable domain were detected using Change-O toolkit (v1.2.0).
Illumina barcodes sequencing data from deep mutational scanning experiments were analyzed using custom scripts (https://github.com/jianfcpku/SARS-CoV-2-RBD-DMS-broad) and Python package dms_variants (v0.8.9).
Custom scripts to analyze the escape mutation profiles data are available at https://github.com/jianfcpku/convergent_RBD_evolution.
Logo plots were generated by Python package logomaker (v0.8) and R package ggseqlogo (v0.1). For unsupervised clustering, we utilized Python package scipy (v1.7.0) and scikit-learn (v0.24.2) to perform multidimensional scaling (MDS), k-means clustering and t-Distributed
Stochastic Neighbor Embedding (t-SNE) embedding. 2D t-SNE plots are generated by ggplot2 (v3.3.3)
Multiple sequence alignments of sarbecovirus RBD were generated using ClustalOmega (v1.2.4)

For manuscripts utilizing custom algorithms or software that are central to the research but not yet described in published literature, software must be made available to editors and reviewers. We strongly encourage code deposition in a community repository (e.g. GitHub). See the Nature Portfolio guidelines for submitting code & software for further information.

Data

Policy information about availability of data

All manuscripts must include a data availability statement. This statement should provide the following information, where applicable:

- Accession codes, unique identifiers, or web links for publicly available datasets
- A description of any restrictions on data availability
- For clinical datasets or third party data, please ensure that the statement adheres to our policy

Processed mutation escape scores can be downloaded at https://github.com/jianfcpku/convergent_RBD_evolution. Sequences and neutralization of the antibodies are included in Supplementary Table 2. Raw sequencing data of DMS assays are available on China National GeneBank (db.cngb.org) with Project accession CNP0003808. We used vdj_GRCh38_alts_ensembl-5.0.0 as the reference of V(D)J alignment, which can be obtained from https://support.10xgenomics.com/single-cell-vdj/software/downloads/latest. We used PDB 6M0J for the structural model of SARS-CoV-2 RBD. The list of strains and the growth advantages were collected from the #24 collection of https://cov-spectrum.org. Designated lineages were from https://github.com/cov-lineages/pango-designation.

Field-specific reporting

Please select the one below that is the best fit for your research. If you are not sure, read the appropriate sections before making your selection.

☒ Life sciences ☐ Behavioural & social sciences ☐ Ecological, evolutionary & environmental sciences

For a reference copy of the document with all sections, see nature.com/documents/nr-reporting-summary-flat.pdf

Life sciences study design

All studies must disclose on these points even when the disclosure is negative.

Sample size	A total of 3333 (3051 cross-reactive+282 Omicron-specific) antibodies were characterized in the manuscript. We analyzed all antibodies in hand and the sample size of antibodies in this study was sufficient to reach statistical significance by two-tailed binomial test for the differences in epitope distribution.<br>Plasma samples were obtained from 40 volunteers who received 3 doses of CoronaVac, 50 BA.1 breakthrough infection convalescent individuals, 39 BA.2 breakthrough infection convalescent individuals, and 36 BA.5 breakthrough infection convalescent individuals who all had received 3 doses of CoronaVac before infection. We analyzed all plasma samples collected and the sample size of plasma could reach statistical significance of NT50 values from neutralization assays by two-tailed Wilcoxon signed-rank test. We also used 4 primary BA.5 infection convalescent individuals' and 3 BA.2 primary infection convalescent individuals' PBMC samples in flow cytometry assay. No sample size calculation was performed.
Data exclusions	846 antibodies were excluded from the study because of insufficient antibody or failed deep mutational scanning experiments, which is defined as no mutations scored two times of the median score.
Replication	Experimental assays were performed in at least two independent experiments according to or exceeding standards in the field. Specifically, we performed mutation screening using two independently constructed mutant libraries. We conducted all neutralization assays and ELISA in at least two independent experiments. All replicates for neutralization and ELISA are successful.
Randomization	Randomization was not required since we were applying a uniform set of measurements across the panel of monoclonal antibodies and plasma. As this is an observational study, randomization is not relevant.
Blinding	Blinding was not required since we were applying a uniform set of measurements across the panel of monoclonal antibodies and plasma. As this is an observational study, investigators were not blinded.

Reporting for specific materials, systems and methods

We require information from authors about some types of materials, experimental systems and methods used in many studies. Here, indicate whether each material, system or method listed is relevant to your study. If you are not sure if a list item applies to your research, read the appropriate section before selecting a response.

Materials & experimental systems

n/a	Involved in the study	
☐	☒	Antibodies
☐	☒	Eukaryotic cell lines
☒	☐	Palaeontology and archaeology
☒	☐	Animals and other organisms
☐	☒	Human research participants
☒	☐	Clinical data
☒	☐	Dual use research of concern

Methods

n/a	Involved in the study	
☒	☐	ChIP-seq
☐	☒	Flow cytometry
☒	☐	MRI-based neuroimaging

Antibodies

Antibodies used	ELISA: 0.25 μg/ml goat anti-human IgG(H+L)HRP (JACKSON, 109-035-003)<br>1 μg/ml H7N9 human IgG1 antibody HG1K (Sino Biologicals, Cat #HG1K) was used as negative control.<br>FACS: The cells were stained with FITC anti-human CD20 antibody (BioLegend, 302304), Brilliant Violet 421 anti-human CD27 antibody (BioLegend, 302824), PE/Cyanine7 anti-human IgM antibody (BioLegend, 314532), PE/Cyanine7 anti-human IgD antibody(BioLegend, 348210).<br>All human antibodies were expressed using Expi293F™ (Gibco, A14527)with codon-optimized cDNA and human IgG1 constant regions in house. The detailed sequence could be found in Supplementary material.
Validation	In this manuscript, we tested 3333 (3051 cross-reactive+282 Omicron-specific) human IgG1 antibodies. All antibodies were expressed using Expi293F™ with codon-optimized cDNA and human IgG1 constant regions. All antibodies' species and specificity to RBD were validated by ELISA. All antibodies neutralization ability was verified by VSV-based pseudotyped virus assays. Details and sequences for all SARS-CoV-2 antibodies evaluated in this study is included in Supplementary Table.<br>Goat anti-human lgG(H+L)HRP (JACKSON, 109-035-003): Based on immunoelectrophoresis and/or ELISA, the antibody reacts with whole molecule human lgG. It also reacts with the light chains of other human immunoglobulins. No antibody was detected against non-immunoglobulin serum proteins. The antibody may cross-react with immunoglobulins from other species.<br>FITC anti-human CD20 antibody was validated by successful staining and FC analysis according to the manufacturer's website https://www.biolegend.com/en-us/products/fitc-anti-human-cd20-antibody-558 and previous publication: Mishra A, et al. 2021. Cell 184(13):3394-3409.e20<br>Brilliant Violet 421 anti-human CD27 antibody was validated by successful staining and FC analysis according to the manufacturer's website https://www.biolegend.com/en-us/products/brilliant-violet-421-anti-human-cd27-antibody-7276 and previous publication Dugan HL, et al. 2021. Immunity. 54(6):1290-1303<br>PE/Cyanine7 anti-human lgM antibody was validated by successful staining and FC analysis according to the manufacturer's website https://www.biolegend.com/en-us/products/pe-cyanine7-anti-human-igm-antibody-12467 and previous publication: Shehata L, et al 2019. Nat Commun. 10:1126<br>PE/Cyanine7 anti-human lgD antibody was validated by successful staining and FC analysis according to the manufacturer's website https://www.biolegend.com/en-us/products/pe-cyanine7-anti-human-igd-antibody-6996 and previous publication: Ahmed R et al. 2019. Cell. 177(6):1583-1599.

Eukaryotic cell lines

Policy information about cell lines

Cell line source(s)	Monoclonal antibody expression: Expi293F™ (Gibco, A14527);<br>Yeast display: EBY100 (ATCC MYA-4941);<br>Pseudutyped virus neutralization assay: Huh-7 (JCRB 0403) ;<br>293T(ATCC, CRL-3216);
Authentication	No authentication was performed beyond manufacturer standards;
Mycoplasma contamination	Not tested for mycoplasma contamination;
Commonly misidentified lines<br>(See ICLAC register)	No commonly misidentified cell lines were used in the study.

Human research participants

Policy information about studies involving human research participants

Population characteristics	Samples were obtained from 40 volunteers who received 3 doses of CoronaVac, 50 BA.1 breakthrough infection convalescent individuals, 39 BA.2 breakthrough infection convalescent individuals, and 36 BA.5 breakthrough infection convalescent individuals who all had received 3 doses of CoronaVac before infection. PBMC samples from 4 primary BA.5 infection convalescent individuals and 3 BA.2 primary infection convalescent individuals were also used in in flow cytometry assay. Gender, age, vaccination profiles, and sampling time point are described in Supplementary table 1. Breakthrough infection individuals are listed in sheet1, and primary infection (without vaccination) samples are listed in sheet2.<br>For the BA.1 breakthrough infection cohort, we presume all individuals were infected by BA.1 since these individuals were infected during the BA.1 wave in Tianjin, China in Jan 2022. A total of 430 patients were confirmed BA.1-infected and no other lineages were detected by sequencing.<br>For the BA.2 breakthrough infection cohort, we presume all individuals were infected by BA.2 since these individuals were infected during the BA.2 wave in Beijing, China in March-May 2022. Some of them were confirmed with sequencing. Others are epidemiologically linked to the confirmed patients.<br>For the BA.5 breakthrough infection cohort, we presume all individuals were infected by BA.5 since some of these individuals were confirmed with sequencing and others are epidemiologically linked to the confirmed patients.
Recruitment	Patients were recruited on the basis of CoronaVac vaccination, post-vaccination BA.1, BA.2 or BA.5 infection, and primary infection of BA.2/BA.5 without vaccination. The only extrusion criteria used were HIV or other debilitating disease. The time intervals between sampling and hospital admission of BA.5 infection samples are shorter than that of BA.1 and BA.2 convalescents, which may cause potential influence on humoral immune responses.

Ethics oversight	Samples from vaccinees and individuals who had recovered from BA.1, BA.2, or BA.5 infection were obtained under study protocols approved by Beijing Ditan Hospital, Capital Medical University (Ethics committee archiving No. LL-2021-024-02) and the Tianjin Municipal Health Commission, and the Ethics Committee of Tianjin First Central Hospital (Ethics committee archiving No. 2022N045KY). All donors provided written informed consent 485 for the collection of information, the use of blood and blood components, and publication of data generated from this study.

Note that full information on the approval of the study protocol must also be provided in the manuscript.

Flow Cytometry

Plots

Confirm that:

☒ The axis labels state the marker and fluorochrome used (e.g. CD4-FITC).

☒ The axis scales are clearly visible. Include numbers along axes only for bottom left plot of group (a 'group' is an analysis of identical markers).

☒ All plots are contour plots with outliers or pseudocolor plots.

☒ A numerical value for number of cells or percentage (with statistics) is provided.

Methodology

Sample preparation	Whole blood sample were diluted 1:1 with PBS+2% FBS (Gibco) and subjected to Ficoll (Cytiva) gradient centrifugation. Plasma was collected from upper layer. Cells were collected at the interface and further prepared by centrifugation, red blood cells lysis (Invitrogen eBioscience) and washing steps. Samples were stored in FBS (Gibco) with 10% DMSO (Sigma) in liquid nitrogen if not used for downstream process immediately. Cryopreserved PBMCs were thawed in PBS+2% FBS.<br>CD19+ B cells were isolated from PBMCs with EasySep Human CD19 Positive Selection Kit II (STEMCELL, 17854). Every 10^6 B cells in 100 µl solution were stained with 3 µl FITC antihuman CD20 antibody (BioLegend, 302304, clone: 2H7), 3.5 µl Brilliant Violet 421 anti-human CD27 antibody (BioLegend, 302824, clone: O323), 2 µl PE/Cyanine7 anti-human IgM antibody (BioLegend, 314532, clone: MHM-88), 2 µl PE/Cyanine7 anti-human IgD antibody (BioLegend, 348210, clone: IA6-2), 0.13 µg biotinylated SARS-CoV-2 BA.2 RBD protein (customized from Sino Biological) or 0.13 µg biotinylated SARS-CoV-2 BA.5 RBD protein (customized from Sino Biological) conjugated with PE-streptavidin (BioLegend, 405204) or APC-streptavidin (BioLegend, 405207), 0.13 µg SARS-CoV-2 WT biotinylated RBD protein 500 (Sino Biological, 40592-V27H-B) conjugated with Brilliant Violet 605 Streptavidin (BioLegend, 405229). Cells are also labeled with biotinylated RBD conjugated to DNA-oligostreptavidin. Cells were washed twice after 30 minutes incubation on ice. 7-AAD (Invitrogen, 00-6993-50) were used to label dead cells.
Instrument	Moflo Astrios EQ (BeckMan Coulter)
Software	Summit 6.0 (Beckman Coulter) for cell sorting; FlowJo 10.8 for data analysis.
Cell population abundance	BA.2 infecion without history infection: 7AAD-&CD20+/singletes=65.7%, CD27+&IgM-&IgD-/7AAD-&CD20+=19.1%, BA.2-RBD+/CD27+&IgM-&IgD-=0.048%, WT-RBD+/BA.2-RBD+= 19.7%<br><br>BA.2 breakthrough infection : 7AAD-&CD20+/singletes=85.9%, CD27+&IgM-&IgD-/7AAD-&CD20+=20.2%, BA.2-RBD+/CD27+&IgM-&IgD-=0.086%, WT-RBD+/BA.2-RBD+= 71.1%<br>BA.2 breakthrough infection (replicate) : 7AAD-&CD20+/singletes=94.5%, CD27+&IgM-&IgD-/7AAD-&CD20+=31.1%, BA.2-RBD+/CD27+&IgM-&IgD-=0.071%, WT-RBD+/BA.2-RBD+= 58.9%<br><br>BA.5 breathrough infecion: 7AAD-&CD20+/singletes=85.8%, CD27+&IgM-&IgD-/7AAD-&CD20+=16.9%, BA.5-RBD+/CD27+&IgM-&IgD-=0.28%, WT-RBD+/BA.5-RBD+= 68.6%<br>BA.5 breakthrough infection (replicate): 7AAD-&CD20+/singletes=85.3%, CD27+&IgM-&IgD-/7AAD-&CD20+=18.1%, BA.5-RBD+/CD27+&IgM-&IgD-=0.17%, WT-RBD+/BA.5-RBD+= 61.4%<br><br>BA.5 infecion without history infection: 7AAD-&CD20+/singletes=87.2%, CD27+&IgM-&IgD-/7AAD-&CD20+=19.3%, BA.5-RBD+/CD27+&IgM-&IgD-=0.071%, WT-RBD+/BA.5-RBD+= 10.3%
Gating strategy	7-AAD−CD20+CD27+IgM−IgD− SARS-CoV-2 BA.2 RBD+ or BA.5+ cells were sorted. The detailed FSC/SSC gating scheme is showed in Extended Data Figure 4.

☒ Tick this box to confirm that a figure exemplifying the gating strategy is provided in the Supplementary Information.
